# Exchange of Insane Hospital Reports

**Published:** 1844-12-28

**Authors:** 


EXCHANGE OF INSANE HOSPITAL REPORTS.

Mr. J. M. Barnard, of Boston, whose name has been heretofore mentioned in connection with a plan for exchanging the annual and other reports of the institutions for the insane, in this and other countries, with a view to uniformity in the system of management—or, rather, for the purpose of informing each other of discoveries in treatment—has just received about four hundred English reports, which will be distributed among the fifteen institutions of the States. With a little care on the part of medical officers in this country, extra copies of all their official papers might be struck off, so that every institution in Europe could have on its file one of them. By a prompt mutual exchange of these documents the happiest results would accrue, and it is therefore cogently urged by Mr. Barnard upon the consideration of gentlemen having a controlling influence in this matter.—*Bost. Med. and Surg. Journ.*

